# Associations with Methylphenidate Treatment in Emotion Regulation and Skin-Picking Severity in Adolescents with Attention-Deficit/Hyperactivity Disorder: A Clinical Follow-Up Study

**DOI:** 10.3390/jcm15062401

**Published:** 2026-03-21

**Authors:** Merve Yazici, Mehmet Kivrak, Uğur Tekeoğlu, Cicek Hocaoglu

**Affiliations:** 1 Department of Child, and Adolescent Psychiatry, Faculty of Medicine, Recep Tayyip Erdogan University, Rize 53100, Turkey; merve.yazici@erdogan.edu.tr (M.Y.); ugur.tekeoglu@erdogan.edu.tr (U.T.); 2 Department of Biostatistics and Medical Informatics, Faculty of Medicine, Recep Tayyip Erdogan University, Rize 53100, Turkey; 3 Department of Psychiatry, Faculty of Medicine, Recep Tayyip Erdoğan University, Rize 53100, Turkey; cicek.hocaoglu@erdogan.edu.tr

**Keywords:** adolescent, attention-deficit hyperactivity disorder, emotion regulation, impulse, methylphenidate, skin picking

## Abstract

**Objective**: To evaluate changes in emotion regulation, skin-picking disorder (SPD) severity, and repetitive thoughts and behaviors in adolescents with attention-deficit/hyperactivity disorder (ADHD) and comorbid SPD during methylphenidate treatment, and to examine the association between emotion regulation and SPD severity. **Materials and Methods**: This naturalistic follow-up study included 26 adolescents aged 11–17 years with ADHD and comorbid SPD. Participants received methylphenidate and were reassessed after three months. Emotion regulation, SPD severity, and repetitive thoughts and behaviors were assessed at baseline and follow-up using the Difficulties in Emotion Regulation Scale (DERS), Skin Picking Scale–Revised (SPS-R), and Repetitive Thoughts and Behaviors Scale–Child Form (RTBS-CF). Pre–post differences were analyzed using paired-sample tests, and associations were examined using correlation and linear regression analyses. **Results**: Significant reductions were observed in total DERS scores (*p* < 0.001, Cohen’s d = 1.35) and all subscales except non-acceptance (*p* = 0.686, Cohen’s d = 0.08). SPS-R and RTBS-CF scores decreased significantly (both *p* < 0.001, Cohen’s d = 1.79 and 0.91, respectively). Changes in DERS scores were moderately correlated with changes in SPS-R scores (r = 0.554, *p* = 0.003). Changes in emotion regulation were significantly associated with changes in SPD severity, accounting for approximately 31% of the variance in this sample. **Conclusions**: Methylphenidate treatment was associated with significant improvements in emotion regulation and concurrent reductions in skin-picking severity in adolescents with ADHD and comorbid SPD. Given the single-arm, pre–post naturalistic design, these findings should be interpreted as associative and exploratory rather than causal.

## 1. Introduction

Attention-Deficit Hyperactivity Disorder (ADHD), characterized by inattention, hyperactivity, and impulsivity, is one of the most common neurodevelopmental disorders of childhood [1,2]. ADHD is frequently accompanied by various psychiatric comorbidities, which significantly complicate the clinical picture and affect the treatment approach and prognosis [3,4]. The comorbidity rates of body-focused repetitive behaviors (BFRBs), such as SPD, are particularly noteworthy [5,6,7].

Skin-picking (excoriation) disorder (SPD) is a mental disorder characterized by recurrent skin picking that leads to tissue damage and clinically significant distress or functional impairment [1]. Along with trichotillomania and onychophagia, it is also defined as a “body-focused repetitive behavior” [8]. In the Diagnostic and Statistical Manual of Mental Disorders (DSM-5), SPD was included as a distinct diagnosis under the category of Obsessive–Compulsive and Related Disorders, along with other disorders like Body Dysmorphic Disorder and Trichotillomania [1]. Its prevalence in the general population is reported to range from 1.4% to 5.4%, and it is often noted to begin during adolescence [8,9,10,11,12]. ADHD comorbidity has been reported as 12.5% in adolescents with SPD [13], and this rate has been shown to reach 8% in adult SPD patients [14]. However, epidemiological studies specifically examining the prevalence of skin-picking disorder within ADHD adolescent populations remain lacking. The pathophysiology and treatment of SPD are still subjects of ongoing debate. Methods such as cognitive behavioral therapy (CBT) and habit reversal training (HRT) have been reported to provide clinical benefit [15,16,17]. Pharmacologically, various options, including selective serotonin reuptake inhibitors (SSRIs), opioid antagonists, glutamatergic agents, antipsychotic augmentation therapies, lamotrigine, and N-acetylcysteine (NAC) have been investigated [17,18,19,20]. However, the response to pharmacological treatment varies significantly among patients, and there is no clear consensus on treatment algorithms.

Various shared mechanisms have been proposed to explain the association between SPD and ADHD, including ectoderm-derived developmental processes, sensory processing dysfunction, impulsivity, and inattention [6,21]. In addition, similar neuroinflammatory mechanisms have been suggested to contribute to both conditions [6]. Among these overlapping features, emotion dysregulation (ED) has been emphasized as a central component in the pathophysiology of both disorders. Emotion regulation refers to the processes through which individuals monitor, evaluate, and modify emotional responses to meet situational demands [22], whereas emotion dysregulation (ED) reflects difficulties in modulating emotional states to support adaptive, goal-directed behavior [23]. Growing evidence indicates that ED plays an important role in both the development and maintenance of SPD [24,25,26]. Skin picking is conceptualized as a maladaptive coping strategy aimed at regulating negative emotional states and may provide transient emotional relief through distraction or soothing sensory input, while ultimately reinforcing and perpetuating the behavior [24,26]. During adolescence—a developmental period characterized by heightened emotional reactivity and still-maturing regulatory capacities—individuals may be particularly prone to engaging in repetitive behaviors as immediate strategies to manage distress [27,28]. In this context, skin picking may function as a short-term strategy to reduce tension or negative affect, even though the temporary relief may reinforce and maintain the behavior through negative reinforcement [8,24]. Consistent with this model, adolescents and adults with SPD have been shown to experience greater difficulties in emotion regulation compared to controls [13,26,29].

In ADHD, ED has been described as a prominent clinical feature by some authors and has been reported in nearly half of affected children, independent of other psychiatric comorbidities [30,31]. In this context, ED is conceptualized as emotional lability, difficulty regulating heightened emotional responses, or limited capacity to generate adaptive positive emotional states [31,32].

Given the substantial clinical and neurobiological overlap between ADHD and SPD—particularly with respect to impulsivity and emotion dysregulation—it has been proposed that pharmacological treatments targeting these domains may also influence comorbid SPD symptoms. Methylphenidate, a first-line treatment for ADHD, enhances attention, impulse control, and executive functioning through inhibition of dopamine and norepinephrine reuptake in fronto-striatal circuits [33,34], and has also been shown to exert beneficial effects on emotion regulation [35,36,37]. By increasing dopaminergic and noradrenergic signaling—particularly within prefrontal and fronto-striatal networks implicated in inhibitory control—methylphenidate may strengthen top-down regulation of urges and habitual responding [33,34,38]. Given evidence that body-focused repetitive behaviors, including SPD, are linked to deficits in inhibitory control and fronto-striatal circuitry [39,40,41], modulation of these catecholaminergic pathways provides a plausible mechanistic rationale for potential changes in skin-picking symptoms during ADHD treatment. On this basis, methylphenidate may be associated with improvements in SPD symptoms in individuals with ADHD by modulating underlying impulsivity and emotion dysregulation.

However, empirical data on this relationship remain extremely limited and are largely derived from case reports [6]. While some reports describe improvement in SPD symptoms following methylphenidate treatment [42,43], others document new-onset or exacerbation of skin-picking behaviors [44,45]. These inconsistent findings underscore the need for systematic studies using quantitative measures. Accordingly, the present study aimed to evaluate changes in SPD symptom severity before and after methylphenidate treatment in adolescents with ADHD and to examine the relationship between these changes and improvements in emotion regulation.

## 2. Materials and Methods

### 2.1. Participants and Procedure

**Figure 1:**
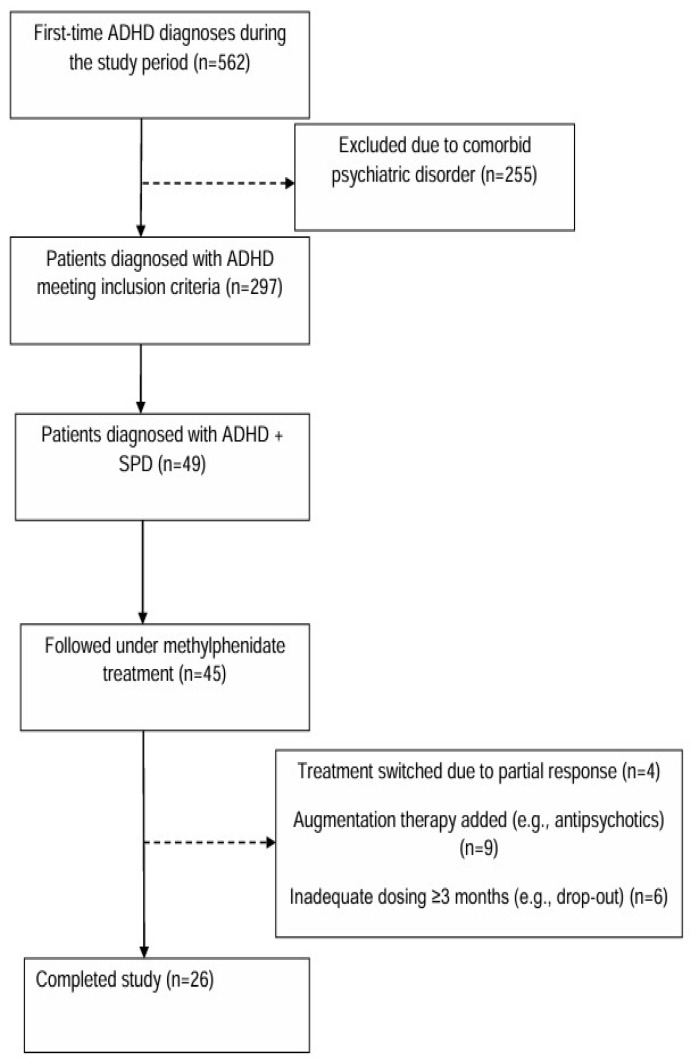
The patient flowchart.

A total of 26 patients, selected from cases who applied to the Child and Adolescent Mental Health and Diseases clinic of Recep Tayyip Erdoğan University Training and Research Hospital between January 2023 and November 2024, were included in this study. These patients, adolescents aged 11–17, who presented to the hospital with inattention, hyperactivity, and academic problems, were diagnosed with ADHD according to DSM-5 criteria based on clinical evaluation, developmental history, teacher reports, and psychometric tests, and had comorbid skin-picking disorder (SPD) symptoms. Since the number of cases was restricted by excluding other comorbid psychopathologies and including only cases with SPD comorbidity in adolescents diagnosed with ADHD, a compromise power analysis was used regarding the number of cases in the clinic. Assuming a Type I error rate (α) of 0.05, a statistical power (1–β) of 0.80, and an effect size of 0.7, the minimum required sample size was calculated as 26 for the regression analysis. Under the same assumptions, the minimum sample size required for two dependent matched pairs was 19 [46]. The detailed power analysis is presented in Supplementary Figure S1. Inclusion criteria for the study were as follows: having a diagnosis of ADHD and SPD, applying due to ADHD symptoms, having been initiated on medium- or long-acting methylphenidate for ADHD treatment, using an effective dose of methylphenidate for at least 3 months, and having an IQ score of 80 or above. During the three-month follow-up period, participants did not receive structured psychotherapy (e.g., cognitive behavioral therapy or habit reversal training), family-based interventions, or formal school-based programs specifically targeting SPD or emotion regulation. The exclusion criteria for the study were as follows: having a diagnosis of autism spectrum disorder, bipolar disorder, major depressive disorder, OCD and related disorders, or schizophreniform disorder, the presence of a chronic disease, and using medication other than methylphenidate. The patient flow chart is shown in Figure 1. Written and verbal consents were obtained from the participants and their families who agreed to be included in the study. A socio-demographic data form created by the researchers included information on age, gender, parental education levels, and class level, and the DSM-5-based Screening and Assessment Scale for Attention-Deficit and Disruptive Behavior Disorders were administered to the parents. The participants were administered the Skin Picking Scale-Revised (SPS-R), which provides information on the severity and frequency of SPD, the DSM-5 Level 2 Repetitive Thoughts and Behaviors Scale-Child Form (RTBS-CF), which was included to assess repetitive thoughts and behaviors beyond skin picking specifically and to provide complementary information regarding broader repetitive phenomena, and the Difficulties in Emotion Regulation Scale (DERS), which evaluates accompanying difficulties in emotion regulation, during the initial assessment and for a second time at the third month of effective dose methylphenidate treatment. The period before methylphenidate treatment was designated as T_1_, and the third-month follow-up visit was designated as T_2_.

All participants were evaluated for psychiatric comorbidities by a child and adolescent psychiatrist using the Schedule for Affective Disorders and Schizophrenia for School-Age Children-Present and Lifetime Version-Turkish Adaptation (K-SADS-PL-DSM-5-T). The K-SADS-PL-DSM-5-T is a comprehensive assessment tool that is administered by an interviewer and allows for evaluation in 23 different diagnostic areas [47].

### 2.2. Assessment Tool

Screening and Assessment Scale for Attention-Deficit and Disruptive Behavior Disorders Based on DSM-5: This scale was developed by Atilla Turgay for the screening of disruptive behavior disorders based on DSM-5 diagnostic criteria. It consists of nine items measuring attention deficit, six items measuring hyperactivity, three items measuring impulsivity, eight items measuring oppositional defiant disorder, and 15 items measuring conduct disorder [48]. The validity and reliability study of this scale was conducted by Ercan et al. [49]. The scale was completed by families and teachers, and ADHD diagnosis and clinical follow-ups were also used.

Skin Picking Scale-Revised (SPS-R): The severity of skin-picking symptoms was assessed using the Skin Picking Scale–Revised (SPS-R), which evaluates the frequency, intensity, time spent, controllability, emotional distress, interference, avoidance, and skin damage associated with skin-picking behaviors. The scale consists of eight items, with the first four items forming the symptom-severity subscale and the remaining four items forming the impairment subscale. Each item is scored on a 5-point Likert scale (0–4), with higher scores indicating greater severity of skin picking [50]. The Turkish version of the SPS-R has previously been used in thesis studies conducted in adult populations [51,52]. However, no published validation study had been conducted in adolescent populations. Prior to its use in the present clinical follow-up study, the Turkish version underwent a preliminary psychometric evaluation in an independent sample (see Supplementary Material: Psychometric Properties of the Turkish Version of the SPS-R). In that independent evaluation, the SPS-R demonstrated acceptable internal consistency (Cronbach’s α = 0.84), and structural validity analyses indicated marginal-to-acceptable model fit indices. These psychometric findings were derived from the independent validation sample and should not be interpreted as having been established within the present clinical follow-up cohort. Given that this psychometric evaluation was conducted in a limited sample and has not yet been independently replicated in adolescents, these findings should be considered preliminary. Further validation studies in larger and independent adolescent samples are warranted. In the present clinical sample, the total SPS-R score was used in subsequent analyses to evaluate changes in skin-picking symptom severity. Detailed psychometric analyses are provided in the Supplementary Material.

The Repetitive Thoughts and Behaviors Scale-Child Form (RTBS-CF) (DSM-5 Level 2): This is an adapted, five-item version of the Florida Obsessive Compulsive Inventory, used to assess repetitive thoughts and behaviors in children and adolescents. It was used in DSM-5 field studies conducted with children between the ages of 11 and 17. The scale is self-administered, and for each item the children are asked to rate the severity of their repetitive thoughts and behaviors over the past 7 days. It consists of five items, and the total score ranges from 0 to 20. Higher scores indicate greater symptom severity. The total score is also divided by five to calculate the mean score. The Turkish validity and reliability study for the scale was conducted by Sapmaz et al. [53].

The Difficulties in Emotion Regulation Scale (DERS): This is a 36-item scale developed by Gratz and Roemer to measure difficulties in emotion regulation [54]. The 5-point Likert-type scale evaluates emotion regulation across six subscales: lack of awareness of emotional responses (awareness); lack of clarity of emotional responses (clarity); non-acceptance of emotional responses (non-acceptance); limited access to effective strategies (strategies); difficulties in controlling impulsive behavior when experiencing negative affect (impulse); and difficulties in engaging in goal-directed behavior when experiencing negative affect (goals). As difficulties in emotion regulation increase, the total score of the scale increases. The Turkish psychometric evaluation of the scale for adolescents was conducted by Sarıtaş et al. [55]. While validated in Turkish adolescent samples, responses from younger adolescents should be interpreted with consideration of developmental variability in emotional awareness.

Approval for the study was obtained from the Recep Tayyip Erdoğan University Interventional Clinical Research Ethics Committee (Ethics Committee approval date: 12 May 2022 and Approval number: 2022/125), and all the procedures involving human participants in the study were in compliance with the ethical standards of the institutional and/or national research committee, and with the 1964 Helsinki Declaration and its later amendments.

### 2.3. Statistical Analysis

Continuous variables were described using mean ± standard deviation (SD) and median (min–max), while categorical variables were summarized as frequency and percentage (%). The difference between pre-treatment (T_1_) and post-treatment (T_2_) measurements was assessed using a paired samples t-test, and Cohen’s d effect size was calculated for each variable. The assumption of normality was verified using the Shapiro–Wilk test. Although subgroup sample sizes were relatively small, parametric analyses were retained because diagnostic checks indicated that the assumptions of the models were reasonably satisfied. For the paired pre–post comparisons, simple difference scores (T2 − T1) were used. For correlation and regression analyses, change scores were calculated as percentage change relative to baseline using the formula (T2 − T1)/T1 × 100) in order to standardize individual differences in baseline severity. Because ratio-based change scores may be sensitive to baseline variability and may increase heteroscedasticity, particularly in small samples, the results derived from these analyses should be interpreted cautiously. Furthermore, simple linear regression analysis was used to investigate the extent to which changes in DERS total and subscale scores explained the change in SPS-R scores. Model fit was reported using the coefficient of determination (R^2^) and 95% confidence intervals. Homoscedasticity was assessed through residual plots. Multicollinearity diagnostics indicated acceptable levels, with variance inflation factor (VIF) values within recommended limits. Bonferroni correction was applied to using the GAMLj linear regression module in Jamovi (v4.2.3). The internal consistency of the SPS-R scale was evaluated using Cronbach’s alpha coefficient. Confirmatory Factor Analysis (CFA) was performed to assess the construct validity of the scales, and model fit was evaluated using RMSEA, SRMR, and CFI indices. Data analysis was conducted using Jamovi 2.4.6 and R (v4.2.3) software. A significance level of *p* < 0.05 was adopted for all tests.

## 3. Results

**Figure 2:**
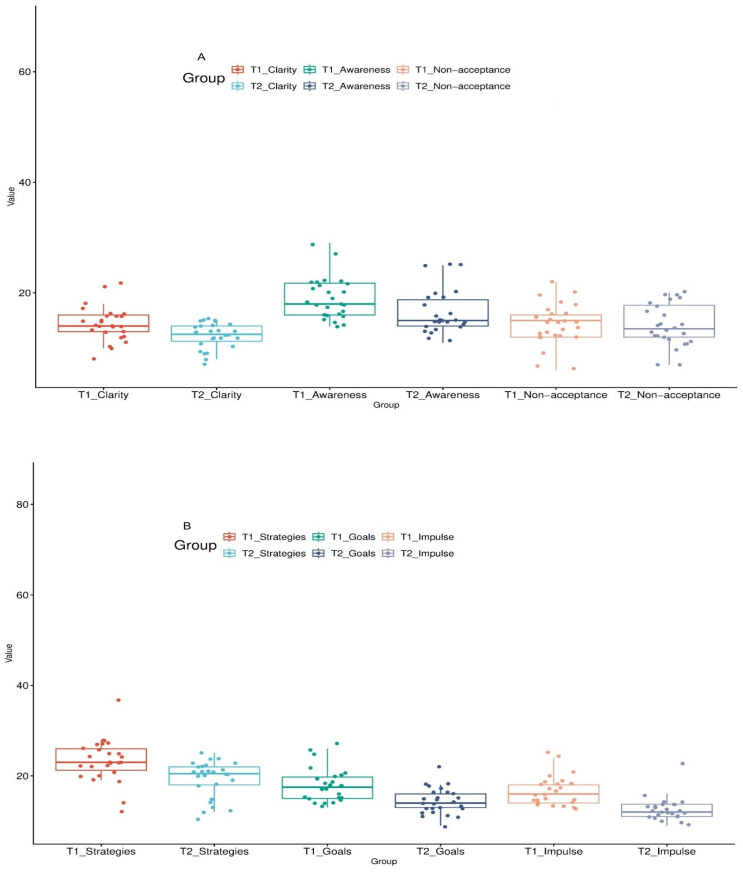
Comparison of DERS subscale scores at baseline (T1) and after treatment (T2). (**A**) Clarity, Awareness, and Non-acceptance subscale scores at baseline (T1) and post-treatment (T2). (**B**) Strategies, Goals, and Impulse subscale scores at baseline (T1) and post-treatment (T2). Boxplots display the median and interquartile range (IQR), with whiskers indicating the minimum and maximum values. Dots represent individual participants. T1 indicates pre-treatment assessment and T2 indicates post-treatment assessment.

A total of 26 adolescents, 10 females and 16 males, were included in the study. The mean age of the cases was found to be 13.2 ± 1.69 years (minimum: 11–maximum: 17.1). The daily median dose of methylphenidate was determined as 33.0 mg/day (IQR: 27–36). The minimum and maximum doses were observed in the range of 18 mg and 72 mg, respectively, and the data showed a moderate distribution with a standard deviation of 11.8. When comparing the patients’ measurements before and after methylphenidate treatment, significant differences were observed in the data for DERS, SPS-R, and RTBS-CF scale scores presented in Table 1 and Figure 2. The total DERS score (t = 6.87, *p* < 0.001) and the scores on its subscales, Clarity (t = 3.376, *p* = 0.002), Awareness (t = 3.862, *p* < 0.001), Strategies (t = 5.185, *p* < 0.001), Goals (t = 5.507, *p* < 0.001), and Impulse (t = 5.822, *p* < 0.001) were found to be significantly lower in the second evaluation after treatment compared to the measurements done before the treatment. However, the difference between groups was not statistically significant in the Non-acceptance subscale (t = 0.409, *p* = 0.686). Similarly, SPS-R (t = 9.118, *p* < 0.001), SPS-R symptom severity (t = 8.45, *p* < 0.001), SPS-R impairment severity (t = 6.99, *p* < 0.001), and RTBS-CF (t = 4.620, *p* < 0.001) scale scores were also found to be significantly lower compared to pre-treatment results.

Exploratory sex-stratified pre–post analyses were conducted to examine clinical changes separately in males and females. Given the small subgroup sizes, detailed results are presented in Supplementary Table S1.

**Figure 3:**
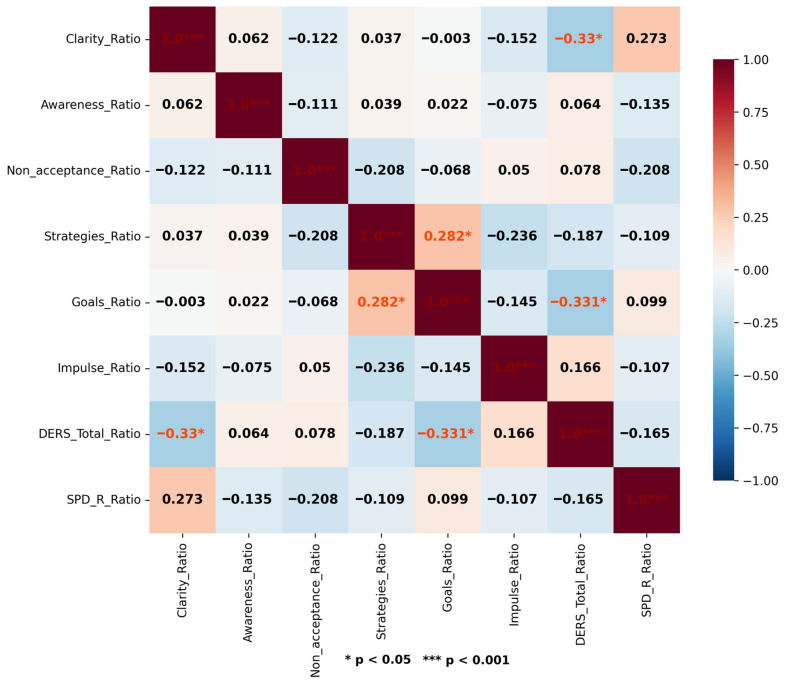
Correlation Matrix of the Study Variables. Pearson correlation coefficients are shown in each cell. Asterisks indicate statistical significance (* *p* < 0.05, *** *p* < 0.001). Darker colors represent stronger correlations.

A moderate positive and statistically significant correlation was found between DERS_Ratio and SPS-R_Ratio (r = 0.554, *p* = 0.003). When the relationship between changes in DERS subscales and SPS-R_Ratio was examined, moderate, positive, and statistically significant correlations were found with changes in the impulsivity dimension (Impulse_Ratio) (r = 0.475, *p* = 0.014) and changes in the strategies dimension (Strategy_Ratio) (r = 0.409, *p* = 0.038). However, no statistically significant relationship was observed with the other sub-dimensions (Figure 3).

Linear regression analysis showed that the change in the total DERS score (DERS_Ratio) was a statistically significant predictor of the change in the total SPS-R score (SPS-R_Ratio). The model explained approximately 31% of the variance in SPS-R_Ratio alone (R^2^ = 0.307). It was found that a one-unit increase in the DERS_ Ratio score corresponded to a statistically significant 1.21-unit increase in SPS-R_ Ratio (β = 1.21, 95% CI [0.45, 1.98], *p* = 0.003). Furthermore, analyses with DERS sub-dimensions revealed that changes in both the Impulse and Strategies sub-dimensions also significantly predicted the change in SPS-R. Approximately 23% of the variance in SPS-R_ Ratio was explained by Impulse_ Ratio (R^2^ = 0.226). It was determined that a one-unit increase in Impulse _Ratio corresponded to a statistically significant 0.62-unit increase in SPS-R_ Ratio (β = 0.618, 95% CI [0.14, 1.10], *p* = 0.014). Similarly, it was observed that approximately 17% of the variance in SPS-R_ Ratio was explained by DERS-S_ Ratio (R^2^ = 0.167). It was found that a one-unit increase in Strategy _ Ratio was associated with a statistically significant 0.61-unit increase in SPS-R _ Ratio (β = 0.605, 95% CI [0.04, 1.17], *p* = 0.038) (Table 2).

## 4. Discussion

In this study, the associations between methylphenidate treatment and changes in emotion regulation skills, SPD symptom severity, and repetitive thoughts and behaviors were examined in adolescents with comorbid ADHD and SPD. After three months of methylphenidate treatment, significant improvement was observed over time in the DERS total score and most of its subscales, while no significant change was determined in the non-acceptance subscale. In parallel, a significant reduction was also observed over the follow-up period in the severity of SPD, which was assessed by the SPS-R, and in the RTBS-CF scores. Regression analyses revealed that changes in emotion regulation—particularly in impulsivity and regulation strategies—were significantly associated with changes in skin-picking symptom severity in this sample. In the literature, there are a small number of conflicting case reports regarding the emergence, exacerbation, or improvement of SPD after methylphenidate treatment in children with ADHD. The present study represents one of the first investigations to examine the relationship between methylphenidate treatment, changes in emotion regulation, and SPD symptom severity using quantitative measures in a sample with comorbid ADHD and SPD. However, given the naturalistic single-arm design, these findings should be interpreted as preliminary and exploratory rather than indicative of a direct treatment effect.

Our results showed significant improvements in the DERS total score and other DERS subscale scores, with the exception of the non-acceptance subscale. These findings are consistent with previous studies that have reported associations between methylphenidate treatment and improvements in emotion regulation in children with ADHD [36,37,56,57,58]. It is believed that catecholaminergic dysregulation in the prefrontal cortex and anterior cingulate cortex, which plays a role in the core pathophysiology of ADHD, contributes to both attention-behavioral control and emotional reactivity [30,33]. By optimizing dopamine and norepinephrine signaling in these regions, methylphenidate may be associated with strengthening executive functions and inhibitory control, which could be considered a possible neurobiological framework for the observed improvements in emotion regulation [4,59]. A notable finding is that no statistically significant change was detected in the DERS-non-acceptance subscale. This subscale assesses an individual’s tendency to avoid or reject negative emotions and appears to be more related to cognitive-schematic processes. Such processes may be less directly influenced by catecholaminergic modulation, which primarily affects executive and inhibitory control mechanisms. The absence of significant change in this domain may indicate that emotional acceptance represents a distinct facet of emotion regulation that does not necessarily follow the same pattern as other regulatory components. This pattern may suggest that changes observed during the treatment period were more closely aligned with executive-control aspects of emotion regulation than with affective acceptance processes. This finding may also reflect limited statistical power, developmental characteristics of the sample, or measurement-related factors, and therefore should be interpreted with caution.

One of the primary outcomes of our study was the observation of statistically significant reductions in scores measuring the severity of SPD, as assessed by the SPS-R, and in repetitive thoughts and behaviors, as measured by the RTBS-CF, over the three-month follow-up period during methylphenidate treatment. This pattern is consistent with the possibility that changes observed in comorbid SPD symptoms may occur alongside treatment of ADHD, rather than necessarily reflecting a direct pharmacological effect on SPD itself. In the existing literature, pharmacological data on SPD—particularly in the context of comorbid ADHD—remain limited, largely restricted to single-case reports with heterogeneous clinical characteristics. However, several case reports have described reductions in skin-picking behavior in individuals with comorbid ADHD and SPD during methylphenidate treatment [42,43]. These reports include both adult (20–30 mg/day) [42] and pediatric (10 mg/day) cases [43], with remission typically observed within weeks to two months; however, outcomes were based primarily on clinical follow-up or parental report rather than standardized SPD severity measures. In contrast, paradoxical or adverse effects have also been documented. Other pediatric [44] and adolescent [45] cases have described acute onset or worsening of skin picking shortly after treatment initiation or dose escalation (10–15 mg/day), sometimes requiring medication discontinuation or additional pharmacotherapy, again without the use of structured SPD outcome instruments. The present findings extend these observations by providing quantitative data in a clinical sample, using standardized measures over a defined three-month follow-up period, while remaining exploratory in nature given the study design.

It can be hypothesized that methylphenidate treatment, in the context of ADHD, may be associated with reductions in internal restlessness and impulsive urges that are thought to precipitate skin-picking behavior [42]. In addition, the observed decrease in RTBS-CF scores suggests that methylphenidate may be associated with broader effects on repetitive thought and behavior patterns, rather than being limited to skin picking alone. Because the SPS-R specifically measures skin-picking severity, whereas the RTBS-CF captures repetitive thoughts and behaviors across a wider phenomenological range, the concurrent reduction observed in both measures allowed us to examine whether changes were restricted to skin-picking symptoms or reflected a broader shift in repetitive phenomena within this sample. This interpretation is consistent with previous reports indicating that methylphenidate may reduce impulsive behaviors such as aggression or antisocial tendencies [60] and, in some cases, obsessive–compulsive symptoms [61]. Considering that body-focused repetitive behaviors such as SPD and trichotillomania have been associated with inhibitory deficits within fronto-striatal circuits [39,40,41], the observed clinical changes could be discussed in relation to potential modulation of these neural systems; however, this remains speculative given the study design. In this context, improvements in impulse control and behavioral inhibition may be considered potential correlates within changes in ADHD-related domains and repetitive behaviors observed over the follow-up period, although causal mechanisms cannot be determined in the present study.

However, rare adverse findings have also been reported in the literature. For example, new-onset skin-picking behavior has been described in a small number of cases after the initiation of methylphenidate treatment, and potential mechanisms underlying this phenomenon have been discussed [44,45]. In these reports, stimulant treatment was suggested to be temporally associated with the emergence of tic-like motor urges or stereotyped behaviors in susceptible individuals, possibly related to dopaminergic modulation. Previous studies have similarly noted that increases in dopamine levels induced by psychostimulants may, in rare cases, be associated with the emergence of tics or repetitive motor behaviors [62]. Such effects have been attributed to individual neurobiological vulnerability, including a predisposition to tic-like behaviors or dopamine-sensitive stereotypies, rather than representing a uniform response to stimulant treatment. These observations underscore the heterogeneity of clinical responses to stimulant treatment and highlight the importance of cautious interpretation when considering potential mechanisms.

One of the important findings of our study was the demonstration of a significant relationship between improvements in emotion regulation and reductions in skin-picking symptoms observed over the follow-up period during methylphenidate treatment. Changes in overall emotion regulation skills, as reflected by the total DERS score, were strongly associated with changes in SPD severity, as measured by the SPS-R. In particular, improvements in the impulsivity and strategies subscales emerged as key correlates of reductions in skin-picking severity. According to the regression model, changes in emotion regulation skills were associated with approximately 31% of the variance explained in changes in SPD symptom severity within this sample. When examined at the subscale level, improvements in both impulse control and access to adaptive regulation strategies were significantly associated with reductions in SPD severity. Taken together, these findings indicate that changes in emotion regulation and changes in SPD symptoms co-occurred in this sample. One possible explanatory framework is that domains such as impulse control and strategy use may be associated within this observed relationship; however, causal mechanisms cannot be determined within the current study design. Consistent with this interpretation, previous studies have reported marked difficulties in emotion regulation—particularly in impulsivity and strategy use—among children and adults with SPD [13,26,29,63]. In emotion regulation models of SPD, skin picking is conceptualized as a maladaptive coping behavior used to manage negative emotional states [24]. Within this framework, limitations in impulse control and restricted access to adaptive strategies increase vulnerability to repetitive behaviors [24,29]. Furthermore, impairments in response inhibition have also been commonly reported in individuals with SPD [40]. Because effective emotion regulation often depends on the ability to inhibit impulsive reactions, deficits in inhibitory control may constrain the use of adaptive regulation strategies [25]. The present findings are broadly consistent with this theoretical model, although longitudinal and controlled studies are required to clarify the directionality and underlying mechanisms of this association.

Our findings can be interpreted in light of the known neurobiological actions of methylphenidate. Methylphenidate inhibits the reuptake of dopamine and norepinephrine, primarily within the prefrontal cortex and striatal regions [33,34,38]. These neural systems play a central role in executive functioning, impulse control, and the regulation of motivated behavior, processes that are commonly disrupted in ADHD. By enhancing catecholaminergic signaling within these circuits, methylphenidate may support improved inhibitory control and emotional regulation, which could, in turn, be associated with reductions in maladaptive repetitive behaviors such as skin picking. In particular, modulation of fronto-striatal and anterior cingulate networks involved in emotional control and behavioral inhibition has been proposed as a potential mechanism underlying changes in attention, impulsivity, and emotion regulation in ADHD. In addition, dopaminergic modulation within reward-related regions such as the nucleus accumbens may contribute to changes in motivational salience and reinforcement processes that underlie repetitive behaviors. Within this framework, the observed reductions in skin picking behavior may be discussed as potentially occurring in the context of broader changes in regulatory processes, rather than necessarily reflecting a direct pharmacological effect on SPD itself.

The findings of this study should be interpreted in light of several limitations. The relatively small sample size, single-center design, and absence of a control group limit the ability to draw causal inferences. As this was a single-arm, pre–post study, improvements observed following methylphenidate treatment cannot be attributed exclusively to the medication, as natural symptom fluctuation, expectancy effects, or concurrent psychosocial influences may also have contributed. The small sample size also limits statistical power and may affect the stability and generalizability of correlation and regression estimates. Given the small and clinically selected sample, individual variability may influence effect size estimates; therefore, the findings should be interpreted cautiously and considered exploratory until replicated in larger samples. Therefore, effect sizes and explained variance values should be interpreted as sample-specific and exploratory. Although Bonferroni correction was applied, the possibility of Type I error inflation due to multiple comparisons cannot be entirely excluded; therefore, the findings should be interpreted cautiously. In addition, the relatively short follow-up period may be insufficient to determine the long-term stability of the observed changes. Outcomes were based on self-report measures and were assessed in a naturalistic clinical setting without blinded raters, which may introduce response or expectancy bias. Furthermore, ADHD symptom change was not systematically quantified at follow-up using a standardized severity measure, which limits direct evaluation of the proposed pathway linking ADHD-related domains and skin-picking severity. Finally, reductions in skin picking may partly reflect decreased ADHD-related boredom or increased engagement rather than specific improvements in emotion regulation. Accordingly, the present findings should be considered preliminary, and future studies incorporating placebo or active comparator groups and longer follow-up periods are warranted.

Despite these limitations, the study has several notable strengths. SPD remains an understudied condition in child and adolescent psychiatry, and this study contributes to the literature by systematically examining changes in SPD symptoms in the context of ADHD pharmacological treatment. The use of validated quantitative measures to assess both symptom severity and emotion regulation (SPS-R, DERS, RTBS-CF) extends existing case-based evidence with structured clinical data. Methodologically, the application of strict inclusion and exclusion criteria and the confirmation of all diagnoses through a structured clinical interview (K-SADS-PL) enhanced diagnostic reliability and sample homogeneity. The absence of concurrent psychotropic medication use further strengthened the interpretability of the findings. Finally, examining the relationship between changes in emotion regulation and changes in SPD symptom severity using regression analysis provided insight into possible explanatory associations beyond descriptive symptom change alone.

## 5. Conclusions

In conclusion, the findings of this study suggest that methylphenidate treatment in adolescents with ADHD and comorbid skin-picking disorder is associated with reductions in skin-picking behavior, alongside improvements in ADHD-related domains such as impulsivity and emotion regulation during the treatment period. Rather than indicating a direct therapeutic effect on SPD, these results suggest that effective management of ADHD may be accompanied by improvement in comorbid behavioral symptoms when emotion regulation and impulse control are concurrently enhanced.

Importantly, the observed association between improvements in emotion regulation and impulse control and reductions in SPD symptom severity highlights the potential clinical relevance of these domains. From a clinical perspective, the findings suggest that management approaches for SPD may benefit from integrated strategies targeting underlying emotion regulation difficulties and impulsivity, in addition to addressing overt behavioral symptoms. However, controlled studies are required to determine causality and clarify treatment sequencing. Future longitudinal and randomized controlled studies are warranted to further elucidate the mechanisms through which methylphenidate may influence comorbid repetitive behaviors.

## Figures and Tables

**Table 1:** Comparison of DERS Subscales, SPS-R, and RTBS-CF Scores Before and After Methylphenidate Treatment.

Variable	T_1_ (Mean ± SD)	T_2_ (Mean ± SD)	Min–Max (Before)	Min–Max (After)	Test Statistics *	*p*- Value	Cohen’s d	95% CI
DERS subcategories								
Clarity	14.42 ± 3.13	12.23 ± 2.34	8–22	7–15	3.376	0.002	0.66	[0.21, 1.11]
Awareness	19.04 ± 3.74	16.42 ± 3.90	14–29	11–25	3.862	<0.001	0.76	[0.30, 1.22]
Non-acceptance	14.35 ± 3.75	14.12 ± 3.81	6–22	7–20	0.409	0.686	0.08	[−0.30, 0.46]
Strategies	23.35 ± 4.81	19.27 ± 4.12	12–37	10–25	5.185	<0.001	1.02	[0.54, 1.50]
Goals	18.00 ± 3.76	14.38 ± 2.82	13–27	9–22	5.507	<0.001	1.08	[0.59, 1.57]
Impulse	16.65 ± 3.25	12.69 ± 2.62	13–25	9–23	5.822	<0.001	1.14	[0.64, 1.64]
DERS Total	106 ± 12.8	89.1 ± 10.6	84–144	66–111	6.87	<0.001	1.35	[0.83, 1.87]
SPS-R	14.73 ± 3.89	7.38 ± 3.77	9–23	3–21	9.118	<0.001	1.79	[1.20, 2.38]
RTBS-CF	4.92 ± 4.22	2.58 ± 2.91	0–15	0–10	4.620	<0.001	0.91	[0.44, 1.38]

*: Paired samples t-test was used. Effect sizes are reported as Cohen’s d for paired samples (d = t/√n), with 95% confidence intervals (CI). DERS: Difficulties in Emotion Regulation Scale; SPS-R: Skin Picking Scale–Revised; RTBS-CF: Repetitive Thoughts and Behaviors Scale–Child Form.

**Table 2:** Predictive effect of changes in DERS total and subscales on SPS-R changes.

			95% Confidence Interval				
Predictor	β	SE	Lower	Upper	R^2^	t	*p*-Value
Intercept	−31.26	6.75	−45.192	−17.33		−4.63	<0.001
DERS_Total_ Ratio	1.21	0.372	0.445	1.98	0.307	3.26	0.003
Intercept	−36.163	6.426	−49.425	−22.9		−5.63	<0.001
Impulse_Ratio	0.618	0.234	0.136	1.1	0.226	2.65	0.014
Intercept	−39.745	6.075	−52.284	−27.21		−6.54	<0.001
Strategies_Ratio	0.605	0.276	0.0357	1.17	0.167	2.19	0.038

Separate linear regression analyses were conducted. Estimates represent unstandardized regression coefficients (β) with standard errors (SE) and 95% confidence intervals (CI). R^2^ indicates the proportion of variance explained by each model. DERS: Difficulties in Emotion Regulation Scale; SPS-R: Skin Picking Scale–Revised. To address the risk of Type I error due to multiple testing across DERS subscales and outcome measures, Bonferroni correction was applied to *p*-values. In addition, regression analyses examining associations between changes in emotion regulation and skin-picking severity were performed using the GAMLj linear regression module in Jamovi, which provides generalized linear modeling within the R framework. The normality of difference scores and regression residuals was evaluated using the Shapiro–Wilk test, and no substantial deviations from normality were observed. Homoscedasticity was assessed through residual plots. Multicollinearity diagnostics indicated acceptable levels, with variance inflation factor (VIF) values within recommended limits (1.02–1.35).

## Data Availability

The datasets used and/or analyzed during the current study are available from the corresponding author on reasonable request.

## Supplementary Materials

The following supporting information can be downloaded at: https://www.mdpi.com/article/10.3390/jcm15062401/s1, Supplementary Table S1: Sex-stratified pre–post comparisons of clinical outcomes following methylphenidate treatment. Supplementary Figure S1: A priori power analysis for the regression and paired-sample analyses. Suplementary material: Psychometric Properties of the Turkish Version of the SPS-R. The Turkish validity and reliability of the SPS-R were established by the authors prior to its use in the present study, as no previous validation studies had been conducted in children and adolescents. For a minimum acceptable Cronbach’s alpha of 0.65, an expected Cronbach’s alpha of 0.80, a significance level of 0.05, a power (1 − β) of 0.80, a number of items (k) of 10, and an expected dropout rate of 10%, the minimum sample size was calculated as 58 [64]. Bartlett test was applied for the adequacy of correlation and was found to be significant (χ^2^ = 323.6; *p* < 0.001). As a result, there were high correlations between the variables, and our dataset was found to be suitable for factor analysis [65]. RMSEA was 0.02, and SRMR was determined as 0.063. RMSEA and SRMR indices range from 0 to 1, and lower values indicate a better model fit [66]. It is stated that an RMSEA value of less than 0.08 and close to 0.06 can be evaluated as a good fit [66,67]. According to conventional criteria (CFI ≥ 0.90), this value may be interpreted as indicating a marginal fit rather than an optimal fit [68].

## Abbreviations

The following abbreviations are used in this manuscript:

ADHD	Attention-Deficit/Hyperactivity Disorder
CBT	Cognitive Behavioral Therapy
DERS	Difficulties in Emotion Regulation Scale
HRT	Habit Reversal Training
RTBS-CF	Repetitive Thoughts and Behaviors Scale-Child Form
SPD	Skin-Picking Disorder
SPS-R	Skin Picking Scale-Revised
SSRIs	Selective Serotonin Reuptake Inhibitors

## References

[1] American Psychiatric Association (2013). Diagnostic and Statistical Manual of Mental Disorders.

[2] Danielson M.L., Claussen A.H., Bitsko R.H., Katz S.M., Newsome K., Blumberg S.J., Kogan M.D., Ghandour R. (2024). ADHD Prevalence Among U.S. Children and Adolescents in 2022: Diagnosis, Severity, Co-Occurring Disorders, and Treatment. J. Clin. Child Adolesc. Psychol..

[3] Faraone S.V., Asherson P., Banaschewski T., Biederman J., Buitelaar J.K., Ramos-Quiroga J.A., Rohde L.A., Sonuga-Barke E.J.S., Tannock R., Franke B. (2015). Attention-Deficit/Hyperactivity Disorder. Nat. Rev. Dis. Primers.

[4] Faraone S.V., Banaschewski T., Coghill D., Zheng Y., Biederman J., Bellgrove M.A., Newcorn J.H., Gignac M., Al Saud N.M., Manor I. (2021). The World Federation of ADHD International Consensus Statement: 208 Evidence-Based Conclusions about the Disorder. Neurosci. Biobehav. Rev..

[5] Kaya Erdogan H., Fıdan S.T., Bulur I., Karapınar T., Saracoglu Z.N. (2017). Evaluation of Cutaneous Findings in Children and Adolescents with Attention Deficit Hyperactivity Disorder: A Preliminary Study. Pediatr. Dermatol..

[6] Loos E., Sekar S., Rosin C., Navarini A.A., Schwale C., Schaefert R., Müller S. (2025). The Relationship Between Chronic Pruritus, Attention-Deficit/Hyperactivity Disorder, and Skin Picking-A Case Series and Narrative Review. J. Clin. Med..

[7] Cheng Y., Lu J.-W., Wang J.-H., Loh C.-H., Chen T.-L. (2023). Associations of Atopic Dermatitis with Attention Deficit/Hyperactivity Disorder and Autism Spectrum Disorder: A Systematic Review and Meta-Analysis. Dermatology.

[8] Grant J.E., Odlaug B.L., Chamberlain S.R., Keuthen N.J., Lochner C., Stein D.J. (2012). Skin Picking Disorder. Am. J. Psychiatry.

[9] Hayes S.L., Storch E.A., Berlanga L. (2009). Skin Picking Behaviors: An Examination of the Prevalence and Severity in a Community Sample. J. Anxiety Disord..

[10] Keuthen N.J., Koran L.M., Aboujaoude E., Large M.D., Serpe R.T. (2010). The Prevalence of Pathologic Skin Picking in US Adults. Compr. Psychiatry.

[11] Grant J.E., Dougherty D.D., Chamberlain S.R. (2020). Prevalence, Gender Correlates, and Co-Morbidity of Trichotillomania. Psychiatry Res..

[12] Ricketts E.J., Snorrason Í., Kircanski K., Alexander J.R., Thamrin H., Flessner C.A., Franklin M.E., Piacentini J., Woods D.W. (2018). A Latent Profile Analysis of Age of Onset in Pathological Skin Picking. Compr. Psychiatry.

[13] Kamberoğlu Turan I., Turan S. (2023). Emotion Regulation and Executive Functions in Adolescents with Skin Picking Disorder. Appl. Neuropsychol. Child.

[14] Grant J.E., Chamberlain S.R. (2022). Characteristics of 262 Adults with Skin Picking Disorder. Compr. Psychiatry.

[15] Schumer M.C., Bartley C.A., Bloch M.H. (2016). Systematic Review of Pharmacological and Behavioral Treatments for Skin Picking Disorder. J. Clin. Psychopharmacol..

[16] Selles R.R., McGuire J.F., Small B.J., Storch E.A. (2016). A Systematic Review and Meta-Analysis of Psychiatric Treatments for Excoriation (Skin-Picking) Disorder. Gen. Hosp. Psychiatry.

[17] Lochner C., Roos A., Stein D.J. (2017). Excoriation (Skin-Picking) Disorder: A Systematic Review of Treatment Options. Neuropsychiatr. Dis. Treat..

[18] Arnold L.M., Auchenbach M.B., McElroy S.L. (2001). Psychogenic Excoriation. Clinical Features, Proposed Diagnostic Criteria, Epidemiology and Approaches to Treatment. CNS Drugs.

[19] Roi C., Bazzano A. (2017). Improvement in Excoriation (Skin-Picking) with Use of Risperidone in a Patient with Developmental Disability. Pediatr. Rep..

[20] Deepmala, Slattery J., Kumar N., Delhey L., Berk M., Dean O., Spielholz C., Frye R. (2015). Clinical Trials of N-Acetylcysteine in Psychiatry and Neurology: A Systematic Review. Neurosci. Biobehav. Rev..

[21] Redden S.A., Leppink E.W., Grant J.E. (2016). Body Focused Repetitive Behavior Disorders: Significance of Family History. Compr. Psychiatry.

[22] Gross J.J. (2002). Emotion Regulation: Affective, Cognitive, and Social Consequences. Psychophysiology.

[23] Thompson R.A. (1994). Emotion Regulation: A Theme in Search of Definition. Monogr. Soc. Res. Child Dev..

[24] Roberts S., O’Connor K., Bélanger C. (2013). Emotion Regulation and Other Psychological Models for Body-Focused Repetitive Behaviors. Clin. Psychol. Rev..

[25] Alexander J.R., Houghton D.C., Bauer C.C., Lench H.C., Woods D.W. (2018). Emotion Regulation Deficits in Persons with Body-Focused Repetitive Behavior Disorders. J. Affect. Disord..

[26] Barber K.E., Fitzgerald J.M. (2025). Emotion Regulation Deficits in Skin Picking (Excoriation) Disorder: A Systematic Review. J. Affect. Disord..

[27] Casey B.J., Jones R.M., Hare T.A. (2008). The Adolescent Brain. Ann. N. Y. Acad. Sci..

[28] Hare T.A., Tottenham N., Galvan A., Voss H.U., Glover G.H., Casey B.J. (2008). Biological Substrates of Emotional Reactivity and Regulation in Adolescence During an Emotional Go-Nogo Task. Biol. Psychiatry.

[29] Snorrason Í., Smári J., Ólafsson R.P. (2010). Emotion Regulation in Pathological Skin Picking: Findings from a Non-Treatment Seeking Sample. J. Behav. Ther. Exp. Psychiatry.

[30] Shaw P., Stringaris A., Nigg J., Leibenluft E. (2014). Emotion Dysregulation in Attention Deficit Hyperactivity Disorder. Am. J. Psychiatry.

[31] Barkley R.A., Fischer M. (2010). The Unique Contribution of Emotional Impulsiveness to Impairment in Major Life Activities in Hyperactive Children as Adults. J. Am. Acad. Child Adolesc. Psychiatry.

[32] Bunford N., Evans S.W., Wymbs F. (2015). ADHD and Emotion Dysregulation Among Children and Adolescents. Clin. Child Fam. Psychol. Rev..

[33] Arnsten A.F.T., Pliszka S.R. (2011). Catecholamine Influences on Prefrontal Cortical Function: Relevance to Treatment of Attention Deficit/Hyperactivity Disorder and Related Disorders. Pharmacol. Biochem. Behav..

[34] Rubia K., Alegria A.A., Cubillo A.I., Smith A.B., Brammer M.J., Radua J. (2014). Effects of Stimulants on Brain Function in Attention-Deficit/Hyperactivity Disorder: A Systematic Review and Meta-Analysis. Biol. Psychiatry.

[35] Fernandez-Pujals A.M., Adams M.J., Thomson P., McKechanie A.G., Blackwood D.H.R., Smith B.H., Dominiczak A.F., Morris A.D., Matthews K., Campbell A. (2015). Epidemiology and Heritability of Major Depressive Disorder, Stratified by Age of Onset, Sex, and Illness Course in Generation Scotland: Scottish Family Health Study (GS:SFHS). PLoS ONE.

[36] Sanabra M., Gómez-Hinojosa T., Grau N., Alda J.A. (2022). Deficient Emotional Self-Regulation and Sleep Problems in ADHD with and without Pharmacological Treatment. J. Atten. Disord..

[37] Kondi K., Takács M., Kovács-Posta E., Szajli C., Sebők-Welker T., Réthelyi J.M., Bunford N. (2025). Emotion Dysregulation in Adolescents Is Normalized by ADHD Pharmacological Treatment. Borderline Personal. Disord. Emot. Dysregul..

[38] Dipasquale O., Martins D., Sethi A., Veronese M., Hesse S., Rullmann M., Sabri O., Turkheimer F., Harrison N.A., Mehta M.A. (2020). Unravelling the Effects of Methylphenidate on the Dopaminergic and Noradrenergic Functional Circuits. Neuropsychopharmacology.

[39] Chamberlain S.R., Blackwell A.D., Fineberg N.A., Robbins T.W., Sahakian B.J. (2005). The Neuropsychology of Obsessive Compulsive Disorder: The Importance of Failures in Cognitive and Behavioural Inhibition as Candidate Endophenotypic Markers. Neurosci. Biobehav. Rev..

[40] Barber K.E., Lee H.-J. (2025). Executive Functioning in Trichotillomania and Skin Picking Disorder: A Review of Neurocognitive Findings. J. Obs.-Compuls. Relat. Disord..

[41] Grant J.E., Odlaug B.L., Chamberlain S.R. (2011). A Cognitive Comparison of Pathological Skin Picking and Trichotillomania. J. Psychiatr. Res..

[42] Bernardes C., Mattos P., Nazar B.P. (2018). Skin Picking Disorder Comorbid with ADHD Successfully Treated with Methylphenidate. Braz. J. Psychiatry.

[43] Çolak Sivri R., Çolak B. (2019). Cessation of Skin Picking Symptoms With Methylphenidate Treatment in a Child With Comorbid Skin Picking and Attention-Deficit/Hyperactivity Disorder. Clin. Neuropharmacol..

[44] Kara T., Akaltun İ. (2018). Newly Developed Skin Picking After Methylphenidate Treatment in Attention Deficit Hyperactivity Disorder: Possible Mechanisms. Clin. Neuropharmacol..

[45] Bensaid N.A., Kisra H. (2025). Onset of Dermatillomania after Treatment with Methylphenidate in a Child with ADHD: A Case Report. Sch. J. Med. Case Rep..

[46] Arslan A.K., Yaşar Ş., Çolak C., Yoloğlu S. (2018). WSSPAS: An Interactive Web Application for Sample Size and Power Analysis with R Using Shiny. Biostat. Biom..

[47] Unal F., Oktem F., Cetin Cuhadaroglu F., Cengel Kultur S.E., Akdemir D., Foto Ozdemir D., Cak H.T., Unal D., Tiras K., Aslan C. (2019). Reliability and Validity of the Schedule for Affective Disorders and Schizophrenia for School-Age Children-Present and Lifetime Version, DSM-5 November 2016-Turkish Adaptation (K-SADS-PL-DSM-5-T). Turk. J. Psychiatry.

[48] Turgay A. (1994). Disruptive Behavior Disorders Child and Adolescent Screening and Rating Scales for Children, Adolescents, Parents and Teachers.

[49] Ercan E.S., Amado S., Somer O., Çıkoğlu S. (2001). Dikkat Eksikliği Hiperaktivite Bozukluğu ve Yıkıcı Davranım Bozuklukları Için Bir Test Bataryası Geliştirme Çabası. Çocuk Gençlik Ruh Sağlığı Derg..

[50] Snorrason I., Olafsson R.P., Flessner C.A., Keuthen N.J., Franklin M.E., Woods D.W. (2013). The Skin Picking Impact Scale: Factor Structure, Validity and Development of a Short Version. Scand. J. Psychol..

[51] Meydan M.S. (2021). Genç Yetişkinlerde Deri Yolma Davranışları Ile Anksiyete ve Obsesif Kompulsif Belirtiler Arasındaki Ilişkinin Incelenmesi. Master’s Thesis.

[52] Dal N. (2021). Genç Yetişkinlerde Deri Yolma Davranışlarıyla Ebeveynlerine Bağlanma Arasındaki Ilişki. Master’s Thesis.

[53] Sapmaz Ş., Erkuran H., Aydemir Ö., Grubu D. (2017). Validity and Reliability of the Turkish Version of DSM-5 Level 2 Repetitive Thoughts and Behaviors Scale-Child Form. Anadolu Psikiyatri Derg..

[54] Gratz K.L., Roemer L. (2004). Multidimensional Assessment of Emotion Regulation and Dysregulation: Development, Factor Structure, and Initial Validation of the Difficulties in Emotion Regulation Scale. J. Psychopathol. Behav. Assess..

[55] Sarıtaş-Atalar D., Gençöz T., Özen A. (2015). Confirmatory Factor Analyses of the Difficulties in Emotion Regulation Scale (DERS) in a Turkish Adolescent Sample. Eur. J. Psychol. Assess..

[56] Ventura P., de Giambattista C., Trerotoli P., Cavone M., Di Gioia A., Margari L. (2022). Methylphenidate Use for Emotional Dysregulation in Children and Adolescents with ADHD and ASD: A Naturalistic Study. J. Clin. Med..

[57] Suzer Gamli I., Tahiroglu A.Y. (2018). Six Months Methylphenidate Treatment Improves Emotion Dysregulation in Adolescents with Attention Deficit/Hyperactivity Disorder: A Prospective Study. Neuropsychiatr. Dis. Treat..

[58] Fernández de la Cruz L., Simonoff E., McGough J.J., Halperin J.M., Arnold L.E., Stringaris A. (2015). Treatment of Children with Attention-Deficit/Hyperactivity Disorder (ADHD) and Irritability: Results from the Multimodal Treatment Study of Children with ADHD (MTA). J. Am. Acad. Child Adolesc. Psychiatry.

[59] Brancati G.E., Acierno D., Barbuti M., Elefante C., Gemignani S., Raia A., Perugi G. (2023). Revisiting Stimulant Use for Emotional Dysregulation in Attention-Deficit/Hyperactivity Disorder (ADHD). Expert. Rev. Neurother..

[60] Kaplan S.L., Busner J., Kupietz S., Wassermann E., Segal B. (1990). Effects of Methylphenidate on Adolescents with Aggressive Conduct Disorder and ADHD: A Preliminary Report. J. Am. Acad. Child Adolesc. Psychiatry.

[61] Rösler M., Retz W., Fischer R., Ose C., Alm B., Deckert J., Philipsen A., Herpertz S., Ammer R. (2010). Twenty-Four-Week Treatment with Extended Release Methylphenidate Improves Emotional Symptoms in Adult ADHD. World J. Biol. Psychiatry.

[62] Madruga-Garrido M., Mir P., Martino D., Cavanna A.E. (2013). Chapter Sixteen—Tics and Other Stereotyped Movements as Side Effects of Pharmacological Treatment. International Review of Neurobiology.

[63] Ricketts E.J., Rozenman M., Snorrason Í., Pérez J.B., Peng M.G., Kim J., Piacentini J. (2019). Confirmatory Factor Analysis of the SLEEP-50 Questionnaire in Trichotillomania (Hair-Pulling Disorder) and Excoriation (Skin-Picking) Disorder. Psychiatry Res..

[64] Bonett D.G. (2002). Sample size requirements for testing and estimating coefficient alpha. J. Educ. Behav. Stat..

[65] Hair J.F., Lovric M. (2011). Multivariate data analysis: An overview. International Encyclopedia of Statistical Science.

[66] Hu L., Bentler P.M. (1999). Cutoff criteria for fit indexes in covariance structure analysis: Conventional criteria versus new alternatives. Struct. Equ. Model..

[67] Steiger J.H. (1990). Structural model evaluation and modification: An interval estimation approach. Multivar. Behav. Res..

[68] Bentler P.M. (1990). Comparative fit indexes in structural models. Psychol. Bull..

