# Supplementary material for: Associations with Methylphenidate Treatment in Emotion Regulation and Skin-Picking Severity in Adolescents with Attention-Deficit/Hyperactivity Disorder: A Clinical Follow-Up Study
Source: J Clin Med. 2026 Mar 21;15(6):2401. doi: 10.3390/jcm15062401 (PMC13026607; doi:10.3390/jcm15062401)
Supplement: Supplementary file 1 [file jcm-15-02401-s001.zip › Suplemantary Table S1. Male of differences in emotion dysregulation and body-focused repetitive behaviors (1).pdf]

Suplemantary Table S1. Sex-stratified pre–post comparisons of clinical outcomes following methylphenidate treatment. To address potential sex-related differences, additional exploratory analyses were conducted separately for males (n = 16) and females (n = 10). Paired-sample t-tests were used to compare baseline **(T1)** and post-treatment **(T2)** scores within each sex group. Test statistics (t), p-values, Cohen's d effect sizes, and 95% confidence intervals (CI) are reported.

**Variable**		**Male (n=16)**		**95% Confidence Interval**			**Female (n=10)**		**95 % Confidence Interval**	
**DERS subcategories**	**Test Statstics**	**p-value**	**Cohen's d**	**Lower**	**Upper**	**Test Statstics**	**p-value**	**Cohen's d**	**Lower**	**Upper**
Clarity	1.987	0.066	0.4967	-0.0313	1.01	3.42	**0.008**	1.0814	0.2722	1.855
Awareness	2.879	**0.011**	0.7197	0.1578	1.263	2.571	**0.03**	0.8132	0.075	1.519
Non-acceptance	0.374	0.713	0.0936	-0.3991	0.583	0.19	0.853	0.0601	-0.5619	0.679
Strategies	5.381	**< .001**	1.3453	0.65	2.018	2.033	0.073	0.6429	-0.0568	1.314
Goals	4.375	**< .001**	1.0938	0.4577	1.708	3.415	**0.008**	1.0799	0.2712	1.853
Impulse	5.526	**< .001**	1.3814	0.6771	2.063	2.724	**0.023**	0.8613	0.1113	1.578
DERS Total	-56.725	**< .001**	-14.1812	-19.0431	-9.137	3.468	**0.007**	1.0968	0.2832	1.875
SPS-R	11.136	**< .001**	2.7841	1.6738	3.876	3.529	**0.006**	1.116	0.2968	1.899
RTBS-CF	4.331	**< .001**	1.0828	0.4491	1.694	2.002	0.076	0.6332	-0.0645	1.303
