# Supplementary material for: Associations with Methylphenidate Treatment in Emotion Regulation and Skin-Picking Severity in Adolescents with Attention-Deficit/Hyperactivity Disorder: A Clinical Follow-Up Study
Source: J Clin Med. 2026 Mar 21;15(6):2401. doi: 10.3390/jcm15062401 (PMC13026607; doi:10.3390/jcm15062401)
Supplement: Supplementary file 1 [file jcm-15-02401-s001.zip › Suplemeantary Figure S1. Power Analysisi of Regression and Matc Paired Sample (1).pdf]

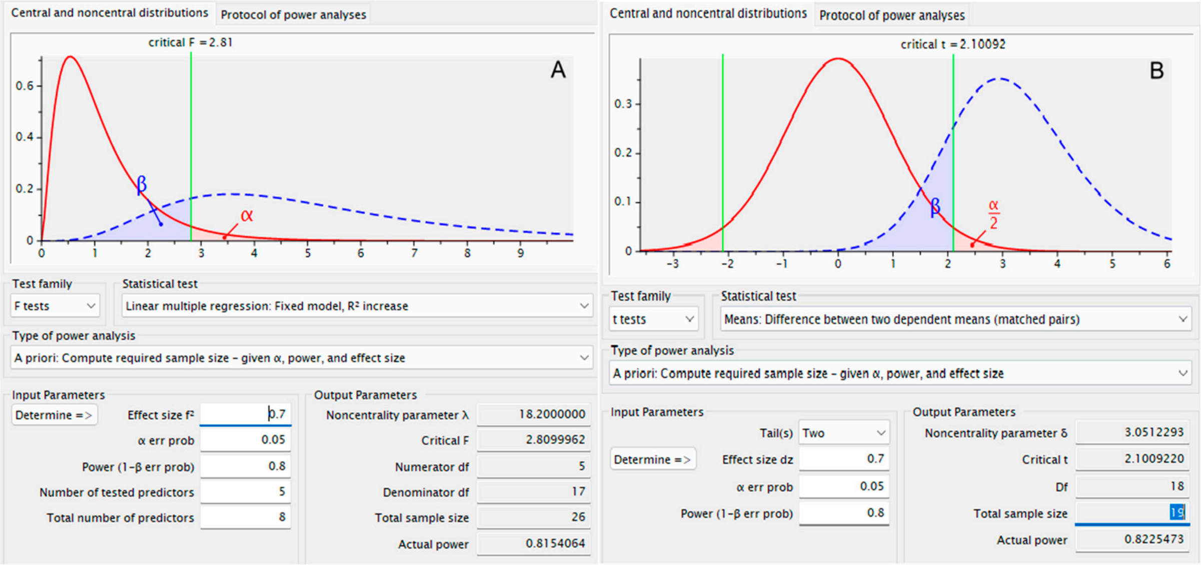

Supplementary Figure S1. A priori power analysis for the regression and paired-sample analyses. Power analyses were conducted using G*Power to justify the sample size required for the primary statistical analyses in this study. Panel **(A)** presents the power analysis for the linear regression model (F-test: linear multiple regression, fixed model, R^2 increase). Assuming a medium-to-large effect size ($f^2 = 0.7$), $\alpha = 0.05$, statistical power $(1 - \beta) = 0.80$, five tested predictors, and eight total predictors, the required minimum sample size was estimated as N = 26. Panel **(B)** shows the power analysis for the paired-sample comparison (two-tailed t-test for dependent means). With an assumed effect size of dz = 0.7, $\alpha = 0.05$, and power = 0.80, the required sample size was calculated as N = 19. The clinical follow-up sample of 26 participants therefore met the minimum required sample size for both the regression and pre–post comparison analyses. The red curves represent the null distributions, the blue dashed curves represent the noncentral distributions under the assumed effect sizes, and the green vertical lines indicate the corresponding critical test statistics.
