# Supplementary material for: Associations with Methylphenidate Treatment in Emotion Regulation and Skin-Picking Severity in Adolescents with Attention-Deficit/Hyperactivity Disorder: A Clinical Follow-Up Study
Source: J Clin Med. 2026 Mar 21;15(6):2401. doi: 10.3390/jcm15062401 (PMC13026607; doi:10.3390/jcm15062401)
Supplement: Supplementary file 1 [file jcm-15-02401-s001.zip › Supplementary Material 1- SPD .pdf]

Supplementary Material : Psychometric Properties of the Turkish Version of the SPS-R

The Turkish validity and reliability of the Skin Picking Scale–Revised (SPS-R) were established by the authors prior to its use in the present study, as no previous validation studies had been conducted in children and adolescents. For a minimum acceptable Cronbach's alpha of 0.65, an expected Cronbach's alpha of 0.80, a significance level of 0.05, a power (1 - β) of 0.80, a number of items (k) of 10, and an expected dropout rate of 10 %, the minimum sample size was calculated as 58 [64]. This number was increased to 65 to account for potential attrition. The validity and reliability of the survey were tested on a total of 79 individuals, exceeding the minimum required sample size (n = 65) calculated for the psychometric analysis, and the retest was conducted on 23 individuals.

In the reliability analysis of the scale, the Cronbach's α internal consistency coefficient was found to be 0.84, indicating that the scale is reliable. The additivity of the scale was examined with the Tukey's Test of Additivity. Whereas the difference between the measurements was statistically significant ($P<0.001$), the non-additivity property was not statistically significant ($P=0.18$). This finding supports the additive nature of the 8-item scale.

In the analysis conducted for the sample size, the KMO test was found to be 0.65. A KMO value greater than 0.50 indicates that the sample size is sufficient. Secondly, the Bartlett test was applied for the adequacy of correlation and was found to be significant ($\chi^2=323.6$; $p<0.001$). As a result, there were high correlations between the variables, and our dataset was found to be suitable for factor analysis [65].

The findings obtained from the structural validity analyses performed using the fit measures of Root Mean Square Error of Approximation (RMSEA), Standardized Root Mean Square Residual (SRMR), and Comparative Fit Index (CFI) showed generally adequate model fit across multiple indices ~~an acceptable fit~~. RMSEA was 0.02, and SRMR was determined as 0.063. RMSEA and SRMR indices range from 0 to 1, and lower values indicate a better model fit [66]. It is stated that an RMSEA value of less than 0.08 and close to 0.06 can be evaluated as a good fit [66.67]. The obtained CFI was determined as 0.841. CFI ranges from 0 to 1, and higher values indicate a better model fit~~. This result indicates that the CFI value is acceptable in terms of fit~~ According to conventional criteria (CFI ≥ 0.90), this value may be interpreted as indicating a marginal fit rather than an optimal fit [68].

Considering the results, it can be stated that the scale is of ~~an acceptable quality~~ adequate psychometric quality. In addition, when the fit of the scale is examined, acceptable model fit observed (χ^2=68.9, $p<0.001$).

The test-retest method was used to determine the reliability of the scale. This method was applied to a total of 23 individuals. Although the number of participants in the test–retest analysis was relatively small, it allowed a preliminary evaluation of temporal stability. The result of the dependent samples t-test performed after the test-retest application showed that there was no statistically significant difference ($p>0.05$).

References

64. Bonett DG. Sample Size Requirements for Testing and Estimating Coefficient Alpha. Journal of Educational and Behavioral Statistics. 2002 Dec 1;27(4):335–40.

65. Hair JF. Multivariate Data Analysis: An Overview. In: International Encyclopedia of Statistical Science [Internet]. Springer, Berlin, Heidelberg; 2011 [cited 2025 Aug 27]. p. 904–7. Available from: https://link.springer.com/rwe/10.1007/978-3-642-04898-2_395

66. Hu L, Bentler PM. Cutoff criteria for fit indexes in covariance structure analysis: Conventional criteria versus new alternatives. Structural Equation Modeling: A Multidisciplinary Journal. 1999 Jan 1;6(1):1–55.

67. Steiger JH. Structural Model Evaluation and Modification: An Interval Estimation Approach. Multivariate Behavioral Research. 1990 Apr 1;25(2):173–80.

68. Bentler PM. Comparative fit indexes in structural models. Psychol Bull. 1990 Mar;107(2):238–46.
